# Apnoeic oxygenation during paediatric intubation: A systematic review

**DOI:** 10.3389/fped.2022.918148

**Published:** 2022-11-21

**Authors:** Shane George, Megan Wilson, Susan Humphreys, Kristen Gibbons, Elliot Long, Andreas Schibler

**Affiliations:** ^1^ Departments of Emergency Medicine and Children’s Critical Care, Gold Coast University Hospital, Southport, QLD, Australia; ^2^ School of Medicine and Menzies Health Institute Queensland, Griffith University, Southport, QLD, Australia; ^3^ Child Health Research Centre, The University of Queensland, Brisbane, QLD, Australia; ^4^ Emergency Department, Tweed Heads Hospital, Tweed Heads, NSW, Australia; ^5^ Emergency Department, Lismore Base Hospital, Lismore, NSW, Australia; ^6^ Department of Anaesthesia, Queensland Children’s Hospital, Brisbane, QLD, Australia; ^7^ Department of Emergency Medicine, The Royal Children’s Hospital, Melbourne, VIC, Australia; ^8^ Clinical Sciences, Murdoch Children’s Research Institute, VIC, Australia; ^9^ Department of Critical Care, University of Melbourne, VIC, Australia; ^10^ Critical Care Research Group, Intensive Care Unit, St Andrews War Memorial Hospital, Brisbane, QLD, Australia; ^11^ Wesley Medical Research, Auchenflower, QLD, Australia

**Keywords:** intubation, paediatric, apnoeic oxygenation, hypoxaemia, safe apnoeic period

## Abstract

**Objective:**

This review assesses the effect of apnoeic oxygenation during paediatric intubation on rates of hypoxaemia, successful intubation on the first attempt and other adverse events.

**Data sources:**

The databases searched included PubMed, Medline, CINAHL, EMBASE and The Cochrane Library. An electronic search for unpublished studies was also performed.

**Study selection:**

We screened studies that include children undergoing intubation, studies that evaluate the use of apnoeic oxygenation by any method or device with outcomes of hypoxaemia, intubation outcome and adverse events were eligible for inclusion.

**Data extraction:**

Screening, risk of bias, quality of evidence and data extraction was performed by two independent reviewers, with conflicts resolved by a third reviewer where consensus could not be reached.

**Data synthesis:**

From 362 screened studies, fourteen studies (*N* = 2442) met the eligibility criteria. Randomised controlled trials (*N* = 482) and studies performed in the operating theatre (*N* = 835) favoured the use of apnoeic oxygenation with a reduced incidence of hypoxaemia (RR: 0.34, 95% CI: 0.24 to 0.47, *p* < 0.001, *I*^2^ = 0% and RR: 0.27, 95% CI: 0.11 to 0.68, *p* = 0.005, *I*^2^ = 68% respectively). Studies in the ED and PICU were of lower methodological quality, displaying heterogeneity in their results and were unsuitable for meta-analysis. Among the studies reporting first attempt intubation success, there were inconsistent effects reported and data were not suitable for meta-analysis.

**Conclusion:**

There is a growing body of evidence to support the use of apnoeic oxygenation during the intubation of children. Further research is required to determine optimal flow rates and delivery technique. The use of humidified high-flow oxygen shows promise as an effective technique based on data in the operating theatre, however its efficacy has not been shown to be superior to low flow oxygen in either the elective anesthetic or emergency intubation situations

**Systematic Review Registration:** This review was prospectively registered in the PROSPERO international register of systematic reviews (Reference: CRD42020170884, registered April 28, 2020).

## Introduction

Infants and children have, in comparison to adults, a lower tolerance for apnoea and become hypoxaemic faster (1). As a result, they are more likely to experience alveolar de-recruitment and significant oxygen desaturation during intubation. Hypoxaemia occurs for 1 in 8 children requiring emergency intubation (2). Previous studies have suggested that hypoxaemia during intubation may increase morbidity and mortality (3, 4).

The provision of apnoeic oxygenation has been proposed to reduce the risk of hypoxaemia during intubation in children. There are two major techniques that could provide apnoeic oxygenation to children undergoing intubation. The adult literature for emergency intubation suggests using 15 L/min of oxygen delivered *via* nasal cannulae during the apnoeic phase, a practice which has been taken up by many adult emergency physicians and has also been adopted in some paediatric institutions (5). Another delivery method using a Transnasal Humidified Rapid-Insufflation Ventilatory Exchange (THRIVE) technique has been suggested to prolong the safe apnoeic time in paediatric and adult patients, thus enabling unhurried intubation in child with an expected difficult airway (6, 7). THRIVE delivers high flow rates (≥2 L/kg/min) of 100% oxygen through specialised nasal cannulae. Its application for intubation in the emergency and critical care environment has not definitively been established.

This systematic review aims to analyse and compare existing studies to help inform clinical practice and to improve patient outcomes. We will assess the effect of using apnoeic oxygenation during the intubation of children aged 0–16 years on rates of hypoxaemia, successful intubation on the first attempt and other intubation related adverse events.

## Methods

### Inclusion criteria

This review has considered studies that include children aged less than 16-years undergoing intubation either for elective or emergent reasons. Studies that evaluate the use of apnoeic oxygenation by any method or device were eligible for inclusion. This review has included studies that report the outcomes: hypoxaemia; number of intubation attempts; time to oxygen desaturation; and intubation related adverse events.

Apnoeic oxygenation is defined as the provision of oxygen through passive insufflation during the apnoeic phase of the intubation attempt.

### Search strategy

The search strategy aimed to find both published and unpublished studies. The databases searched included: PubMed; Medline; CINAHL; EMBASE; and The Cochrane Library. The search for unpublished studies included: Clinical trial registries (Cochrane Central Register of controlled trials and ClinicalTrials.gov) to identify recent and ongoing studies; Google Scholar web search; and bibliographies from included studies, known reviews and text for additional citations.

Detailed search strings and results for each database are included in Supplementary Digital Content S1. The search was undertaken on 16 October 2019, all referenced studies at this date were included for screening.

### Data collection and analysis

#### Selection of studies

Two review authors independently assessed titles and abstracts and, when needed, full texts of identified studies to determine eligibility for inclusion using the Covidence systematic review software (Veritas Health Innovation, Melbourne, Australia). Conflicts were resolved by discussion between reviewers and then by consulting with a third review author where a consensus could not be reached.

#### Data extraction and management

Full-text versions of all studies were obtained, and data extraction was completed using standardised data extraction form by two reviewers independently. Extracted data were compared for any differences, with conflict resolved by discussion between reviewers. Where consensus could not be reached a third reviewer was engaged. Extracted data were exported into Review Manager 5 (8) for processing, comparison and analysis.

#### Assessment of included studies

Risk of bias was assessed according to the Cochrane Collaboration revised tool for assessing the risk of bias for randomised trials (ROB 2) (9) and the Risk Of Bias In Non-Randomized Studies - of Interventions (ROBINS-I) for non-randomised studies (10). The Grading of Recommendations, Assessment, Development, and Evaluation (GRADE) approach (11), was used to assess the quality of evidence for the following clinically relevant outcomes: hypoxaemia, first intubation attempt success rate; first attempt success rate for intubations without hypoxaemia; tracheal intubation related adverse events.

Evidence from RCTs was considered as high-quality but was downgraded by one level for serious (or two levels for very serious) limitations according to the following: design (risk of bias), consistency across studies, directness of evidence, precision of estimates, and presence of publication bias.

### Data analysis

Children included in randomised studies were analysed on an intention-to-treat (ITT) basis. Assuming low levels of heterogeneity, analysed treatment effects in individual trials were initially analysed using a fixed-effect model for meta-analysis of combined data. If substantial heterogeneity was noted and there was no significant asymmetry in the associated funnel plot, analysis was undertaken using a random-effect model and we examined the potential cause of heterogeneity by performing subgroup and sensitivity analyses. If meta-analysis was inappropriate, we analysed and interpreted individual trials separately. The Mantel-Haenszel (MH) method was used for calculating estimates of risk ratio (RR) and are presented along with associated 95% confidence intervals (21, 22). For measured quantities, we used the inverse variance method. Appropriate graphical representation of the meta-analyses have been generated.

Heterogeneity of studies owing to the nature of the interventions provided is anticipated, this was assessed using *χ*^2^ and *I*^2^ statistics. Significant heterogeneity was investigated by performing subgroup analyses.

## Results

### Literature search results

The systematic search returned 362 non-duplicate citations; an additional 6 citations were included in the manual reference check of included papers. After screening of title and abstract, 327 citations were excluded leaving 41 for full text review. A further 27 papers were excluded after full-text review. Fourteen studies remained meeting all inclusion and no exclusion criteria. There were 6 randomised controlled trials, 3 prospective observational study, 3 “before and after” observational studies and 2 retrospective observational studies. One ongoing randomised controlled trial was identified, however no interim data has been published (12).

**Figure 1:**
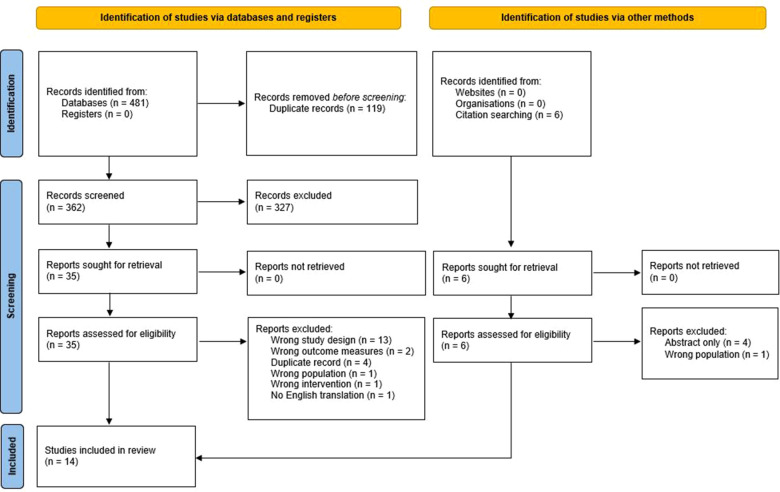
PRISMA 2020 flow diagram.

A PRISMA (Preferred Reporting Items for Systematic Reviews and Meta-Analyses) flow chart is presented in Figure 1.

**Figure 2:**
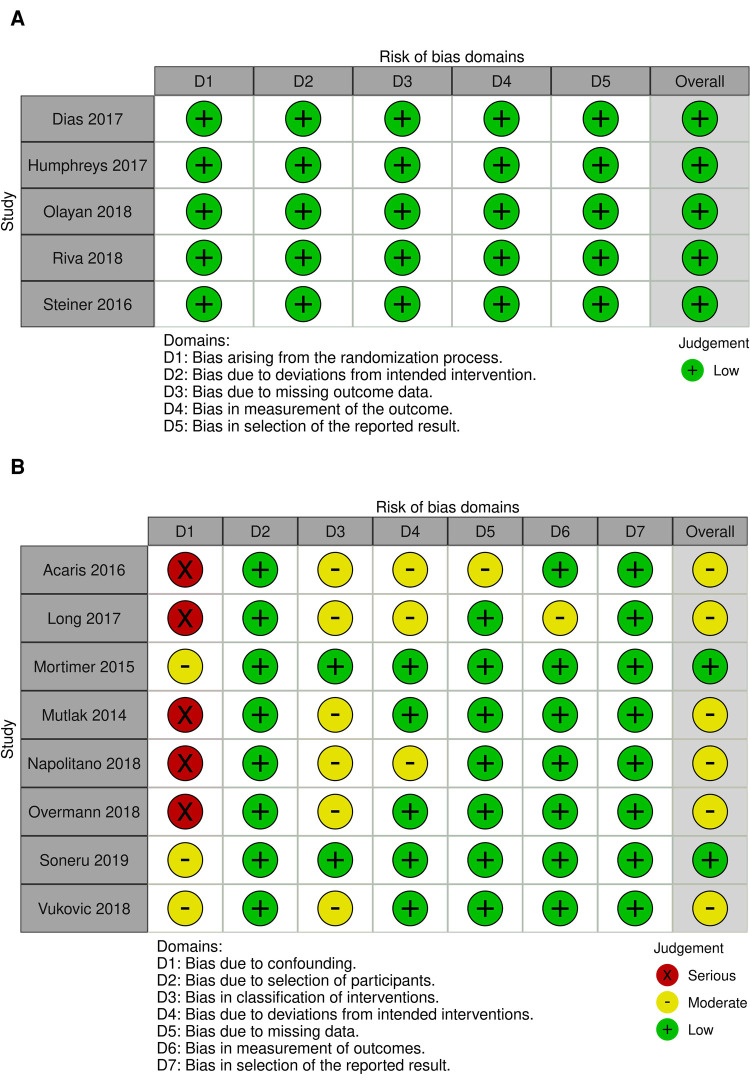
Risk of bias summary: review authors’ judgements about each risk of bias item for each included study. (**A**) Revised Cochrane risk of bias for randomised trails (ROB 2). (**B**) Risk of bias in non-randomised studies of interventions (ROBINS-I).

Included studies provided a total of 2,442 patients for inclusion, with 1,159 children in operating theatre setting and 1,283 children being intubated in the ED or PICU environment. Characteristics of included studies and patient populations is included in Table 1. Risk of bias assessment for included studies is presented in Figure 2.

**Table 1:** Characteristics of included studies.

First Author	Study Design (*n*)	Patient Population	Intervention (*n*/*N*)	Comparator (*n*/*N*)	Primary Outcome	Secondary Outcomes	GRADE of Evidence
Arcaris (2016)	Prospective Cohort (*N* = 93)	<18 years Emergency Dept	15 L/min apnoeic oxygenation (50/93)	No apnoeic oxygenation (43/93)	SpO_2_ < 90%	1. SpO_2_ < 80% 2. SpO_2_ < 70%	Low
Dias (2017)	RCT (*N* = 95)	Full term infants, aged <6 months, Elective or emergency surgery	Apnoeic oxygenation *via* OxiPort™ Miller Laryngoscope at 2 L/min (47/95)	No apnoeic oxygenation using standard Miller laryngoscope (48/95)	Lowest SpO_2_	1. SpO_2_ < 90% 2. SpO_2_ < 85% 3. Correlation between time to intubation and SpO_2_	Moderate
Humphreys (2017)	RCT (*N* = 48)	<10 years old Elective surgery or imaging	Humidified high flow apnoeic oxygenation (THRIVE) (23/48)	No apnoeic oxygenation (24/48)	Time to SpO_2_ < 92%	1. Change in tcCO_2_ 2. Change in heart rate	High
Long (2017)	Observational Cohort study (before and after intervention) (*N* = 117)	<18 years Emergency Dept	Implementation of a quality improvement initiative, including apnoeic oxygenation (46/117)	Standard practice prior to QI initiative implementation (71/117)	First attempt successful intubation without hypoxia or hypotension	Nil	Low
Mortimer (2015)	Prospective Cohort (*N* = 44)	<18 years Emergency Dept or PICU	Apnoeic oxygenation with age based flow rates 5–15 L/min	Nil	SpO_2_ < 80%	Number of intubation attempts	Low
Mutlak (2014)	Retrospective Observational study (*N* = 65)	1–10 kg Elective Surgery	Apnoeic oxygenation *via* TruView Laryngoscope at 2–5 L/min (22/65)	C-MAC (23/65) or Standard Mackintosh (20/65) laryngoscope	Time to intubation	Lowest SpO_2_	Low
Napolitano (2018)	Observational Cohort study (before and after intervention) (*N* = 575)	PICU	Apnoeic oxygenation with age based flow rates 5–15 L/min (291/575)	Infrequent use of apnoeic oxygenation at clinicial discretion (284/575)	SpO_2_ < 80%	1. Feasibility of Apnoeic Oygenation 2. SpO_2_ < 70% 3. TIAEs	Low
Olayan (2018)	RCT (*N* = 30)	1–8 years old with ASA I or II for elective surgery	Apnoeic oxygenation at 3 L/min (15/30)	No apnoeic oxygenation (15/30)	Time to SpO_2_ < 92%	Number of patients with SpO_2_ < 92% and < 95%	High
Overmann (2018)	Retrospective observational Study (*N* = 305)	<18 years Emergency Dept	Apnoeic oxygenation with age based flow rates 2–6 L/min (227/305)	No apnoeic oxygenation (78/305)	SpO_2_ < 90%	1. Time to desaturation 2. Duration of desaturation episode 3. Lowest SpO_2_	Moderate
Riva (2018)	RCT (*N* = 60)	1–6 years and 10–20 kg for elective surgery	Humidifed High flow apnoeic oxygenation (THRIVE) at 2 L/kg/min	1. THRIVE with FiO2 0.3 2. Low flow apnoeic oxygenation at 0.2 L/kg/min	Time until one of: 1. SpO_2_ < 95% 2. Apnoea >10 min 3. tcCO_2_ > 65 mmHg	1. Increase in CO_2_ 2. Adverse Events	High
Soneru (2019)	Prospective Observational Study (*N* = 356)	Age <8 years for elective surgery	Apnoeic oxygenation at 5 L/min	Standard practice, no apnoeic oxygenation	Time to SpO_2_ < 05%	1. SpO_2_ < 95% 2. SpO_2_ < 90% 3. Lowest SpO_2_ 4. Intervention by senior operator required	Moderate
Steiner (2016)	RCT (*N* = 457)	1–17 years old ASA I-III for elective dental procedure under anaesthetic	1. TruView Laryngoscope with apnoeic oxygenation at 2–3 L/min 2. Standard Laryngoscopy with apnoeic oxygenation attached oxygen cannula at 2–3 L/min	No apnoeic oxygenation	Time to a 1% absolute decrease in SpO_2_ from baseline	Rate of desaturation over time	High
Vukovic (2018)	Observational Cohort study (before and after intervention) (*N* = 149)	<22 years of age Emergency Dept	Apnoeic oxygenation with age based flow rates 4–8 L/min (90/149)	No apnoeic oxygenation	SpO_2_ < 90%	1. Lowest SpO_2_ 2. Number of attempts	Low
Windpassinger (2016)	RCT (*N* = 48)	0–2 years, elective surgery, ASA I or II	Airtraq laryngoscope, oxygen at 4 L/min (24/48)	Airtraq Laryngoscopy, no apnoeic oxygenation (24/48)	Time to desaturation after intubation and cessation of apnoeic oxygenation	Time to intubation	High

### Clinical outcome results

#### Oxygen saturation (SpO_2_) < 90%

All studies included a measure of hypoxaemia as a primary or secondary outcome, however there was significant variation in the cut-off value used to define hypoxaemia between studies.

Eight studies (three RCTs, five observational studies) reported the outcome SpO_2_ < 90% as either a primary or secondary outcome with a total of 1,382 patients available for analysis. All RCTs included were conducted in the operating theatre (430 patients). Two observational studies were conducted in the operating theatre (421 patients) with the remaining three studies conducted in an ED (two studies, 398 patients) or PICU (one study, 149 patients). All included studies used oxygen at flow rates of <15 L/min (Table 1).

**Figure 3:**
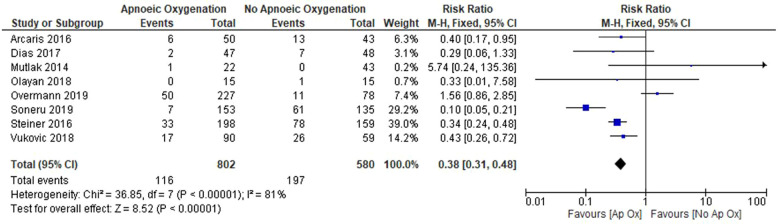
Forrest plot of included studies for outcome of SpO2 < 90% (fixed effect model).

Overall, there was a decreased incidence of hypoxaemia in patients who receive apnoeic oxygenation (RR: 0.38, 95% CI: 0.31 to 0.48, *p* < 0.001); however, the included studies display significant heterogeneity (*I*^2^ = 84%) (Figure 3). Consistent findings were demonstrated when incorporating random effects (RR: 0.42, 95% CI: 0.22 to 0.82, *p* = 0.01) (Supplementary Figure S1).

**Figure 4:**
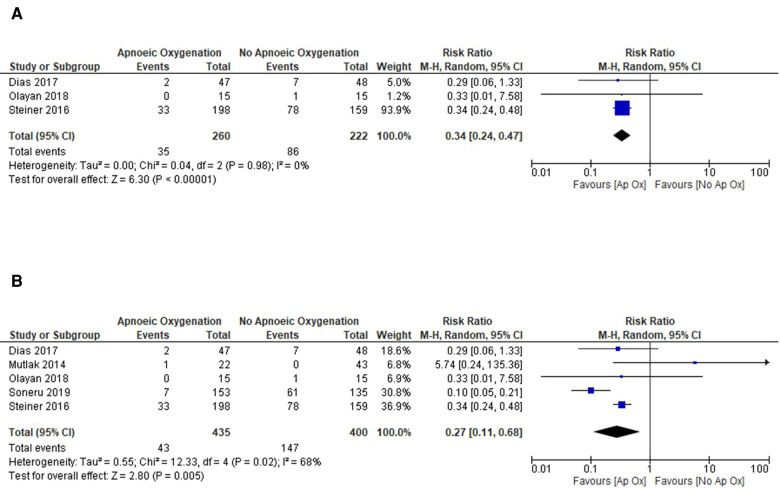
Subgroup analysis of included studies for outcome of SpO_2_ < 90%. (**A**) Forrest plot of included RCTs for outcome of SpO2 < 90%. (**B**) Forrest plot of included studies in the operating theatre setting for outcome of SpO2 < 90%.

Given the high degree of heterogeneity in the included studies, subgroup analysis was undertaken to investigate the effects in the high quality RCT studies and to also compare effects in the different patient groups of elective and emergent admissions. The three RCTs included were all performed on elective surgery patients in the operating theatre, with an ASA of I or II providing a more standardised group of patients at baseline. Meta-analysis of this data shows a reduced incidence of hypoxaemia in the apnoeic oxygenation group (RR: 0.34, 95% CI: 0.24 to 0.47, *p* < 0.001) when compared to control and no heterogeneity between studies (*I*^2^ = 0%) (Figure 4A).

The methodological quality was higher for the majority of studies in the operating theatre and meta-analysis of these studies also showed a significantly reduced risk of hypoxaemia with mild heterogeneity between studies (RR: 0.27, 95% CI: 0.11 to 0.68, *p* = 0.005) (Figure 4B). Studies performed in the ED and PICU were of lower methodological quality and displayed significant heterogeneity in their results and were thus unsuitable for meta-analysis. Given this, it is difficult to estimate the treatment effect in this cohort of patients.

#### Lowest oxygen saturations

Six included studies (1,000 patients) reported the lowest SpO_2_ during intubation as a primary or secondary outcome (13–18). There are conflicting results from these studies and methodological differences and bias make the data unsuitable for meta-analysis.

Four studies reported a small, but statistically significant, difference in the median lowest oxygen saturations between apnoeic oxygenation and control groups (13, 16–18). Three studies favoured the use of apnoeic oxygenation (13, 17, 18); however, all three studies report median/mean SpO_2_ of >95% with small IQR/SD making the clinical significance of this difference questionable. A single study reported a higher SpO_2_ in the no apnoeic oxygenation group, however this finding is difficult to interpret due to baseline differences in SpO_2_ prior to commencement of intubation potentially confounding this outcome (16).

Two studies reported no difference in the median lowest oxygen saturation during intubations when comparing apnoeic oxygenation to control (14, 15). Mutlak et al. report oxygen desaturation in a single patient in their cohort (*N* = 65), however the authors note this is likely due to inadequate preoxygenation. Overmann et al. report no significant difference in lowest SpO_2_, however they only report the lowest SpO_2_ for patients who experience a desaturation to < 90%. Data for the entire cohort is not presented.

#### Number of intubation attempts

Four included studies reported the number of intubation attempts as a primary or secondary outcome (15, 16, 19, 20). All studies report data as part of a departmental quality improvement initiative, one reports data for after the intervention, with no control for comparison (20). The remaining studies report conflicting outcomes, and display high risk of bias.

A study by Long et al. report no change in the first attempt success rate between groups (78% for both groups), however there was a difference in first pass success without hypoxaemia (78% post-intervention, 49% pre-intervention). Importantly all patients in the post-intervention group received apnoeic oxygenation, a significant number of the pre-intervention group (54%, 38/71) also received apnoeic oxygenation during their intubation attempt (19). Data is not presented on the proportion of pre-intervention patients without apnoeic oxygenation as a subgroup analysis. Overmann et al. (15) also report no difference in first pass success in their retrospective cohort. In contrast, Vukovic et al. also report that patients who receive apnoeic oxygenation had higher first attempt success (20/90 and 28/62, *p* = 0.0025) (16).

Given the high risk of bias and methodological variability meta-analysis was not performed.

#### Safe apnea time

Two RCTs have investigated the effect of humidified high flow nasal prong apnoeic oxygenation on the time to oxygenation desaturation. In an RCT comparing humidified high flow apnoeic oxygenation to control in an elective surgery setting Humphreys et al. demonstrated a significant increase (*p* < 0.001) in the time to desaturation across four age cohorts (0–6 months, 6–24 months, 2–5 years and 6–10 years) (21). Riva and colleagues also demonstrated a longer safe apnoea time in children receiving either low flow or humidified high flow apnoeic oxygenation through a composite end-point of hypoxaemia, duration of apnoea and transcutaneous CO_2_ (22).

In addition, two observational studies have reported the time taken for a child to experience hypoxaemia (as measured by a drop in SpO_2_). Soneru reports in a prospective cohort study an increase in the median time taken for a child's oxygen saturation to fall below 95% when apnoeic oxygenation is applied (202 s IQR 142–253 s) compared to no apnoeic oxygenation (127 s, IQR 83–165 s) (18). Conversely, in a retrospective observational study Overmann reported there was no significant difference in the median time to desaturation between the apnoeic oxygenation group (140 s, IQR 108–382 s) and the control group (125 s, IQR 83–272 s, *p* = 0.25) (15).

An alternative measure of time to desaturation was used by Steiner et al. where time to desaturation was defined as the time taken for a 1% absolute decrease in SpO_2_ from the pre-intubation level. The study compared two different methods of apnoeic oxygenation delivery with a control group. When compared to control (30 s, 95% CI: 24–39 s) the time to desaturation was longer for both apnoeic oxygenation groups (67 s, 95% CI: 35–149 s, *p* < 0.001 and 75 s, 95% CI: 37–122 s, *p* < 0.001) (23).

#### Tracheal intubation related adverse events (TIAE)

An observational study by Napolitano et al. reported the rate of TIAE between apnoeic oxygenation and control groups. The rate of adverse events was lower in the in the apnoeic oxygenation cohort when compared to control (6% and 10% respectively), but did not reach statistical significance (*p* = 0.14) (24).

## Discussion

There is an emerging body of literature around methods and application of apnoeic oxygenation during paediatric intubation, as demonstrated by the recency of publication for all included studies in this review. The heterogeneity of methods used to deliver apnoeic oxygenation, differences in characteristics of included patients and lack of consistency in outcome measures make it difficult to directly compare studies or make definitive treatment recommendations. Despite this, the available data from the operating theatre setting for elective procedures suggest a reduced incidence of hypoxemia with the provision of apnoeic oxygenation in this cohort.

Studies included in this review have examined two groups of patients which have significantly different baseline characteristics. One group of studies looks at patients undergoing elective medical procedures, who have no significant underlying medical conditions or pathologies while the other examines patients in the ED or PICU being intubated for a life-threatening critical illness with significant physiological compromise. Direct comparison of these two groups needs to be interpreted with caution and there is likely to be a differential effect of the intervention based on the severity of illness and presence (or absence) of respiratory failure at the time of intubation. Further research is required to better understand this relationship with an adequate sample size to power for this subgroup analysis.

The flow rates used to deliver apnoeic oxygenation are markedly variable between studies, making direct comparison difficult and estimation of the true effect size unreliable. The use of standard nasal cannulae is favoured by many clinicians as they are readily available, inexpensive, and simple to apply. However, to deliver high flow rates specialised high-flow nasal cannulae are required as the diameter of the nasal outlet is too narrow to accommodate higher flows. The use of higher flow rates of at least 2 L/kg/min through the THRIVE technique has gained increasing popularity in anesthesia despite limited understanding of the exact physiological mechanism of its demonstrated effect. To date there have been no studies published investigating the use of the THRIVE technique in the PICU or ED setting, despite promising pilot data for children in the operating theatre (21, 22).

The lack of standardised core outcome measures in airway management literature is a significant barrier to interpretation and application of the available data. The most consistent outcome measures reported are hypoxaemia, although the exact level varies, the number of intubation attempts required to successfully insert the endotracheal tube and the lowest SpO_2_ during the attempt. All of these outcome measures are objective, available and clinically relevant. While we are unlikely to achieve consensus on what level of hypoxaemia is clinically significant, the general standards of reporting mild (SpO_2_ 81%–90%), moderate (SpO_2_ 71%–80%) and severe (SpO_2 _< 70%) hypoxaemia would allow better comparison of the available data.

Some studies included in this review have reported statistically significant differences in the lowest SpO_2_ reported, with the values reported being >95% (13). While there may be a statistically significant difference the in values reported, the clinical significance of these differences is minimal.

The data available on the efficacy of apnoeic oxygenation in the paediatric population is more limited than that in the adult population, however there are similar methodological and physiological issues across both groups. In the adult literature there have been numerous systematic reviews published over recent years (25–30). In these reviews there is a consistent trend towards a reduction in the incidence of hypoxaemia and an increase in the proportion of intubations successful on first attempt but there is a paucity of high quality randomised controlled studies. Similar to the paediatric literature there is a lack of consensus on the optimal method of delivery of apnoeic oxygenation, as well contradictory reports on its efficacy in different patient populations, particularly patients with respiratory failure, and different treatment environments.

## Limitations

This literature review is confined to a relatively small pool of patients (*n* = 2,442) from studies of variable methodological quality and inconsistent outcome reporting. Two of the included studies were abstracts only (24, 31). Despite attempts to contact the authors, more detailed data could not be obtained. The review includes a number of retrospective observational studies limiting their internal validity and generalisability.

## Conclusions

There is a growing body of evidence to support the use of apnoeic oxygenation during the intubation of children. Further research is required to determine optimal flow rates and delivery technique. The use of humidified high-flow oxygen shows promise as an effective technique based on data in the operating theatre, however its efficacy has not been shown to be superior to low flow oxygen in either the elective anesthetic or emergency intubation situations.

High quality randomised controlled trials with standardised outcome measures are required for both elective and emergent intubations, adequately powered for subgroup analysis for age, severity of illness and reason for intubation.

## Data Availability

The original contributions presented in the study are included in the article/Supplementary Material, further inquiries can be directed to the corresponding author/s.
