# Supplementary material for: Apnoeic oxygenation during paediatric intubation: A systematic review
Source: Front Pediatr. 2022 Nov 21;10:918148. doi: 10.3389/fped.2022.918148 (PMC9720125; doi:10.3389/fped.2022.918148)
Supplement: Supplementary file 1 [file Table1.docx]

**Supplemental Digital Content 1: Database search strings**

**MEDLINE:**

(((MH "Pediatrics")) OR (TX ((pediatric OR pediatrics OR paediatric OR paediatrics OR child OR children OR adolescent OR adolescents OR infant OR infants OR neonate OR neonates OR newborn OR newborns)))) AND (TX ((apnoeic oxygenation OR apneic oxygenation OR "transnasal humidified rapid insufflation ventilatory exchange" OR trans-nasal humidified rapid-Insufflation ventilatory exchange OR "transnasal humidified rapid-insufflation ventilatory exchange" OR THRIVE))) AND (((MH "intubation, intratracheal")) OR (TX intubation) OR ((MH "Anesthetics")) OR (TX (anesthetic OR anaesthetic OR anaesthesia OR anesthesia)))

**PubMed:**

Search (((((pediatrics[MeSH Terms]) OR (((((((((((((((pediatric) OR paediatric) OR pediatrics) OR paediatric) OR child) OR children) OR adolescent) OR adolescents) OR infant) OR infants) OR neonate) OR neonates) OR newborn) OR newborns)))) AND (((((apneic oxygenation) OR apnoeic oxygenation) OR "transnasal humidified rapid insufflation ventilatory exchange") OR "transnasal humidified rapid-insufflation ventilatory exchange"))) AND ((((((((airway management[MeSH Terms]) OR intubation[MeSH Terms]) OR anesthesia[MeSH Terms]) OR intubation)) OR airway management)) OR paediatric anaesthesia))

**EMBASE:**

(apneic AND oxygenation OR (apnoeic AND oxygenation) OR 'apneic oxygenation' OR 'apnoeic oxygenation' OR 'transnasal humidified rapid insufflation ventilatory exchange' OR 'transnasal humidified rapid-insufflation ventilatory exchange') AND ('pediatrics' OR 'pediatric'/exp OR pediatric OR 'pediatrics'/exp OR pediatrics OR 'paediatric'/exp OR paediatric OR 'child'/exp OR child OR 'children'/exp OR children OR 'adolescent'/exp OR adolescent OR 'adolescents'/exp OR adolescents OR 'infant'/exp OR infant OR 'infants'/exp OR infants OR 'neonate'/exp OR neonate OR neonates OR 'newborn'/exp OR newborn OR newborns) AND ('respiration control'/exp OR 'respiration control' OR 'airway management'/exp OR 'airway management' OR (('airway'/exp OR airway) AND ('management'/exp OR management)) OR 'intubation'/exp OR 'intubation' OR 'anesthesia'/exp OR 'anesthesia' OR ('paediatric anaesthesia' AND paediatric AND anaesthesia) OR 'pediatric anesthesia' OR (pediatric AND anesthesia) OR anesthetic OR anaesthetic OR anaesthesia OR anesthesia)

**CINAHL:**

(((MH "Pediatrics")) OR (TX ((pediatric OR pediatrics OR paediatric OR paediatrics OR child OR children OR adolescent OR adolescents OR infant OR infants OR neonate OR neonates OR newborn OR newborns)))) AND (TX ((apnoeic oxygenation OR apneic oxygenation OR "transnasal humidified rapid insufflation ventilatory exchange" OR trans-nasal humidified rapid-Insufflation ventilatory exchange OR "transnasal humidified rapid-insufflation ventilatory exchange" OR THRIVE))) AND (((MH "intubation, intratracheal")) OR (TX intubation) OR ((MH "Anesthetics")) OR (TX (anesthetic OR anaesthetic OR anaesthesia OR anesthesia)))

**(a)**


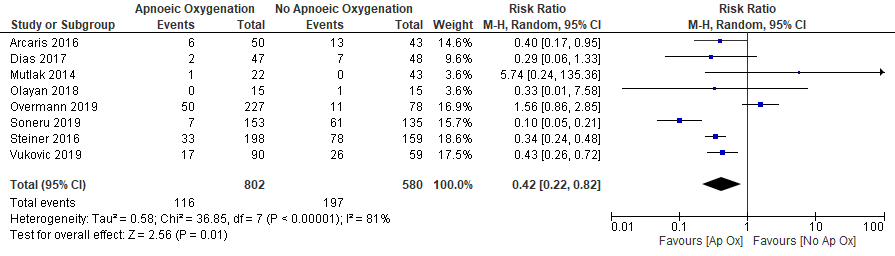


**(b)**


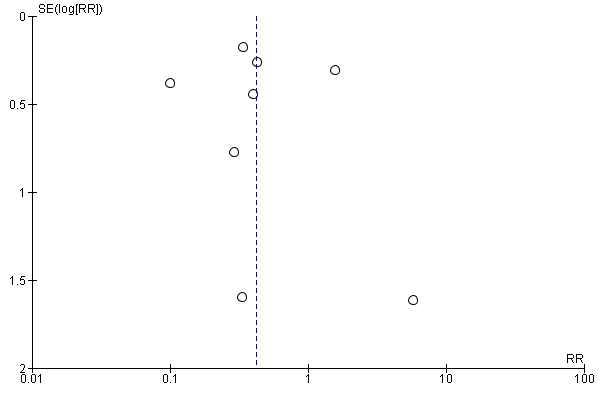


**Supplementary Figure 1:**

**(a)** Forrest plot of included studies for outcome of SpO_2_ <90% (random effect model)

**(b)** Funnel plot of comparison: Apnoeic Oxygenation vs. No Apnoeic Oxygenation for Hypoxaemia (SpO2 < 90%).
